# Educational inequality in the occurrence of abdominal obesity: *Pró-Saúde* Study

**DOI:** 10.1590/S0034-8910.2015049005786

**Published:** 2015-09-23

**Authors:** Ronaldo Fernandes Santos Alves, Eduardo Faerstein

**Affiliations:** I Programa de Pós-Graduação em Saúde Coletiva. Instituto de Medicina Social. Universidade do Estado do Rio de Janeiro. Rio de Janeiro, RJ, Brasil; II Departamento de Epidemiologia. Instituto de Medicina Social. Universidade do Estado do Rio de Janeiro. Rio de Janeiro, RJ, Brasil

**Keywords:** Obesity, Abdominal, epidemiology, Socioeconomic Factors, Health Status Disparities, Gender and Health

## Abstract

**OBJECTIVE:**

To estimate the degree of educational inequality in the occurrence of abdominal obesity in a population of non-faculty civil servants at university campi.

**METHODS:**

In this cross-sectional study, we used data from 3,117 subjects of both genders aged 24 to 65-years old, regarding the baseline of *Pró-Saúde* Study, 1999-2001. Abdominal obesity was defined according to abdominal circumference thresholds of 88 cm for women and 102 cm for men. A multi-dimensional, self-administered questionnaire was used to evaluate education levels and demographic variables. Slope and relative indices of inequality, and Chi-squared test for linear trend were used in the data analysis. All analyses were stratified by genders, and the indices of inequality were standardized by age.

**RESULTS:**

Abdominal obesity was the most prevalent among women (43.5%; 95%CI 41.2;45.9), as compared to men (24.3%; 95%CI 22.1;26.7), in all educational strata and age ranges. The association between education levels and abdominal obesity was an inverse one among women (p < 0.001); it was not statistically significant among men (p = 0.436). The educational inequality regarding abdominal obesity in the female population, in absolute terms (slope index of inequality), was 24.0% (95%CI 15.5;32.6). In relative terms (relative index of inequality), it was 2.8 (95%CI 1.9;4.1), after the age adjustment.

**CONCLUSIONS:**

Gender inequality in the prevalence of abdominal obesity increases with older age and lower education. The slope and relative indices of inequality summarize the strictly monotonous trend between education levels and abdominal obesity, and it described educational inequality regarding abdominal obesity among women. Such indices provide relevant quantitative estimates for monitoring abdominal obesity and dealing with health inequalities.

## INTRODUCTION

Obesity is an important global public health problem, with rising trends in several development contexts.^28^ Obese people have increased risks of adverse outcomes in the long run, and that even holds true for people with no metabolic abnormalities, as compared to individuals of normal weight and metabolically healthy.^17^ In Brazil, around 50.0% of the adult population is overweight, and 15.0% of it is obese.^a^

According to the World Health Organization, obesity regards to abnormal or excessive body fat accumulation.^28^ Even though body mass index has been the classic anthropometric measurement in population studies, abdominal circumference has been observed to have better predictive abilities for certain obesity-related morbidities.^29^ Besides that, as it measures abdominal obesity, it is a key element in the definition of metabolic syndrome^19^ and in the analysis of risks for cardiovascular diseases, diabetes, cancer, and death.^4^ ^,^ ^24^

The relationship between socioeconomic position and obesity is consistent, but it is observed to have variations according to genders and levels of economic development.^5^ ^,^ ^21^ ^,^ ^23^ In low-income countries, a higher probability for obesity is observed among groups of high socioeconomic positions in both genders. In medium and high-income countries, the association between socioeconomic position and obesity is frequently shown to be inverse among women, especially regarding education, whereas it is observed to be both direct and not statistically significant among men. Those changes in the association pattern between socioeconomic position and obesity take place in early economic development stages, thus revealing the importance of studies and preventive interventions in that context.^5^ ^,^ ^7^ ^,^ ^21^ ^,^ ^23^

Measures which are based on the contrasts between comparison groups are commonly used analytic strategies to report the extent and direction of the association between socioeconomic position and obesity. However, they have the simultaneous interpretation of different partial inequality estimates as a disadvantage, as polytomous variables are often used as socioeconomic position indicators.^30^ Alternate methods have been proposed to measure and monitor health-related socioeconomic inequalities, considering only one quantitative estimate of inequalities;^14^ ^,^ ^30^ however, they are not very disseminated in the epidemiological practice.

Even though the relationship between socioeconomic position and obesity is well documented in the epidemiological literature, its association with abdominal obesity, especially, is not yet very understood. Furthermore, no publications were found reporting socioeconomic inequality regarding abdominal obesity in adults in Brazil. Exploring the inequality regarding abdominal obesity may be important to enhance actions to prevent obesity and its consequences. This study intended to estimate the degree of educational inequality in the occurrence of abdominal obesity in a population of non-faculty civil servants at university campi.

## METHODS

In this cross-sectional study, we used data from *Pró-Saúde* Study baseline (EPS* – *1999-2001). EPS is a longitudinal investigation program of non-faculty civil servants at a university campi in Rio de Janeiro, Southeastern Brazil, mainly focusing on social determinants of health.^9^ So far, four data collection stages were conducted (1999, 2001, 2006, and 2012) by trained teams comprising field researchers, supervisors, and coordinators. The EPS baseline was simultaneously composed of eligible subjects in the first two stages.

We included all employees in the permanent staff of the university who accepted to take part in stages 1 and 2 (baseline) of EPS. Pregnant women and people older than 65 years of age were excluded. A pilot study, pre-testing of research instruments and procedures, independent entering of data by two professionals, and monitoring of the collection and data processing ensure the quality of analyzed information.^8^ ^,^ ^9^ Covariates were obtained from 1999 census; independent and dependent variables, from 2001 census.

The concentration of fat in the abdominal region was evaluated by a double measurement of abdominal circumference at the level of the navel, through the use of a 180-meter measuring tape. Subjects, in order to be taken measurements, kept their arms folded over their chests, their feet close together, their weights equally distributed between their legs, their abdomens relaxed, and their breathing at regular paces. Abdominal obesity was defined according to abdominal circumference thresholds of 88 cm for women and 102 cm for men, as suggested by the World Health Organization.^29^

A multidimensional, self-administered questionnaire was used to collect the information on genders (male, female), age in years (24 to 34, 35 to 44, 45 to 54, 55 to 65), and education levels (incomplete elementary education, complete elementary education, incomplete high school education, complete high school education, incomplete university education, complete university education, graduate studies).

Preliminary statistical analyses included: absolute and relative frequencies, prevalence of abdominal obesity, and respective 95% confidence intervals and Chi-squared test for the linear trend among ordinal variables and dichotomous outcome. Slope (SII) and relative (RII) indices of inequality were used to estimate the degree of educational inequality in the occurrence of abdominal obesity. Those indices may produce absolute and relative estimates of the socioeconomic gradient in health, and they are based on weighted regression analysis.^14^ ^,^ ^30^ Linear and logistic regression was used to respectively calculate SII and RII, as well as the respective 95% confidence intervals. The dependent variable was abdominal obesity (dichotomous). The independent variable was a numerical score that was defined from the median of the cumulative interval in each category of the social polytomous variable in the horizontal scale of the population (Table 1). Thus, instead of attributing ordinal values (e.g., 1, 2, 3,..., k, for a series of k categories) to subjects from the respective educational categories, numerical values were attributed – they considered: (a) the information from all simultaneously ordered categories; (b) the proportional size of those categories; and (c) their relative positions within the population scale. Finally, such numerical values were employed in the related regression models by the numerical score. SII and RII were standardized by ages.

**Table 1:** Frequency measurements used in the algebraic proposition of the numerical score.

Ordinal values (variable)	Absolute frequency	Relative frequency	Cumulative frequency	Cumulative relative frequency	Cumulative population interval	Median of the interval (score)
1	f_1_	fr_1_	F_1_	Fr_1_	0.0 - Fr_1_	Score 1
2	f_2_	fr_2_	F_2_	Fr_2_	Fr_1_ |-Fr_2_	Score 2
3	f_3_	fr_3_	F_3_	Fr_3_	Fr_2_ |-Fr_3_	Score 3
...	...	...	...	...	...	...
k	f_k_	fr_k_	F_k_ = N	Fr_k_ = 1.0	Fr_k-1_ |-1.0	Score k

k: (ordinal) index of the social polytomous variable; f_k_: absolute frequency; fr_k_: relative frequency (commonly expressed as %); F_k_: cumulative frequency; Fr_k_: cumulative relative frequency; N: total of subjects in the population

Note: [fr_k_ = f_k_ ÷ N] e [Fr_k_ = F_k_ ÷ N]; [f_1_ = F_1_] e [fr_1_ = Fr_1_].

Below is an algebraic proposition to clarify the numerical score. In Table 1, a logic arrangement of used frequency measurements is shown in matrix format, in order to support reading of the numerical score formulation.



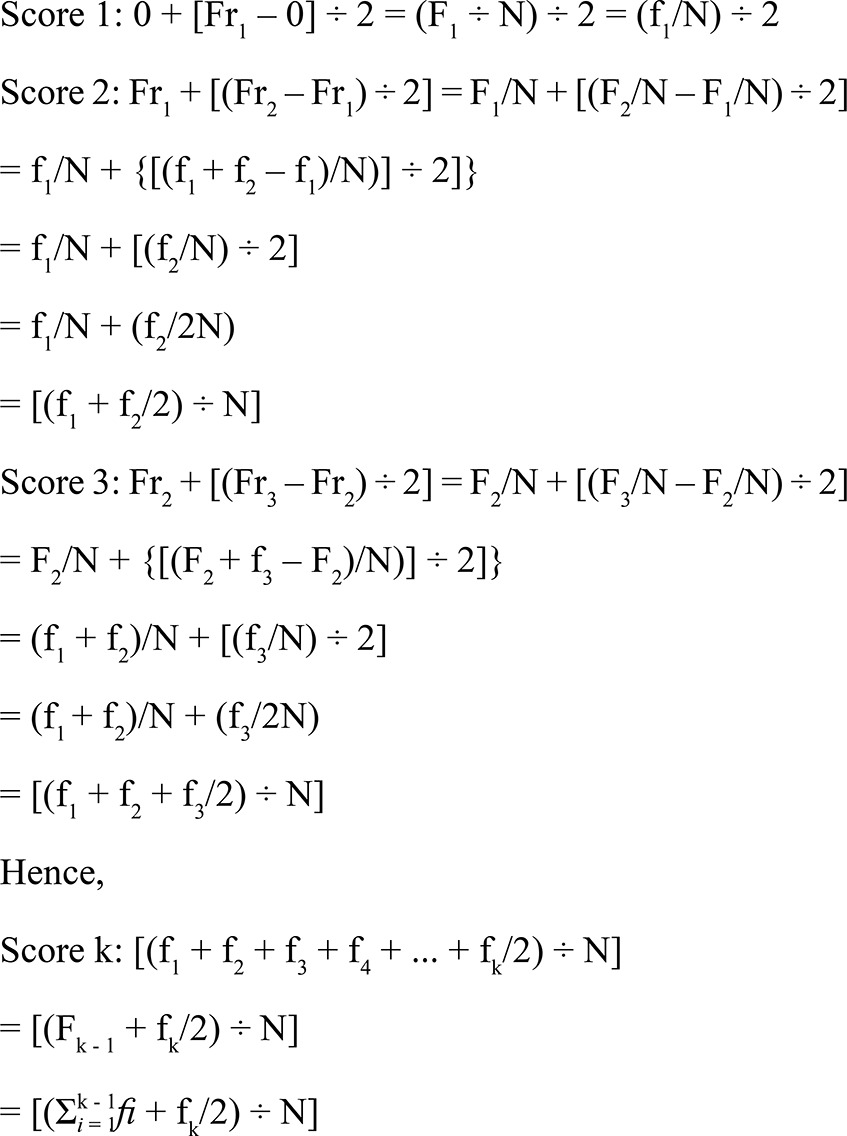



All analyses were stratified by genders and processed in R 3.1.0 software.

The EPS 1999 and 2001 protocols were approved by the Research Ethics Committee of Pedro Ernesto University Hospital, of Universidade do Estado do Rio de Janeiro (Record 224/1999; Record 461/2001). All subjects signed informed consent forms. Data were analyzed in a way to ensure subjects total anonymity.

## RESULTS

The study population comprised subjects who were eligible in the two first stages of EPS 1999 and 2001. During field work, 9.6% of subjects skipped participation in stage 1 – and 16.5% in stage two, which totaled 3,253 subjects in both stages (77.9% of eligible ones), who regard to the baseline of the investigation program. The abdominal perimeters of 52 people could not be measured (1.6%); 21 subjects (0.7%) were excluded the data analysis as they were older than 65 years; 63 subjects (1.9%) did not answer the question regarding education level variable. Finally, a sample of 3,117 adults was obtained (95.8% of subjects).

The women in the sample slightly outnumbered the men (Table 2). The average age was 42.7 (95%CI 42.3;43.1) years for women and 41.2 (95%CI 40.8;41.6) for men. The women were observed to have high educational levels, and 47.0% of them had at least finished university, whereas for men, a share of 36.0% was observed regarding that. The average age was higher in the categories with the lowest education levels, ranging from 53 to 40 years among women, and from 48 to 50 years among men.

**Table 2:** Sociodemographic characteristics in *Pró-Saúde* Study baseline, 1999-2001.

Variable	Women		Men		Total	
	n	%	n	%	N	%
Gender	1,739	55.8	1,378	44.2	3,117	100
Age (years)						
24 to 34	281	16.2	307	22.3	588	18.9
35 to 44	765	44.0	623	45.2	1,388	44.5
45 to 54	541	31.1	367	26.6	908	29.1
55 to 65	152	8.7	81	5.9	233	7.5
Education level						
Incomplete elementary schooling	102	5.9	125	9.1	227	7.3
Complete elementary schooling	90	5.2	97	7.0	187	6.0
Incomplete high school education	132	7.6	151	11.0	283	9.1
Complete high school education	358	20.6	311	22.6	669	21.5
Incomplete university education	235	13.5	204	14.8	439	14.1
Complete university education	478	27.5	321	23.3	799	25.6
Graduate studies	344	19.8	169	12.3	513	16.5

The prevalence of abdominal obesity in the baseline population of EPS was 35.0% (95%CI 33.3;36.7). It was significantly higher among women (43.5%; 95%CI 41.2;45.9) as compared to men (24.3%; 95%CI 22.1;26.7), in all educational strata and age ranges (Table 3). Among the women, the probability for being obese increased with the age, and it was notably higher in the group of 55 to 65-year olds (73.0%; 95%CI 65.2;79,9). Decreased prevalence of abdominal obesity was observed among 55 to 65-year old men, but the small population size of that subgroup may have influenced that specific estimate.

**Table 3:** Prevalence (%) of abdominal obesity, slope index of inequality, and relative index of inequality of the female population. *Pró-Saúde* Study, 1999-2001.

Variable	Women			Men			Total		
	Abdominal obesity (%)	95%CI	p^a^	Abdominal obesity (%)	95%CI	p^a^	Abdominal obesity (%)	95%CI	p^a^
Gender	43.5	41.2;45.9		24.3	22.1;26.7		35.0	33.3;36.7	
Age (years)			< 0.001			< 0.001			< 0.001
24 to 34	26.0	20.9;31.5		16.9	12.9;21.6		21.3	18.0;24.8	
35 to 44	36.3	32.9;39.9		24.1	20.8;27.6		30.8	28.4;33.3	
45 to 54	54.5	50.2;58.8		30.2	25.6;35.2		44.7	41.4;48.0	
55 to 65	73.0	65.2;79.9		27.2	17.9;38.2		57.1	50.4;63.5	
Education level			< 0.001			0.436			< 0.001
Incomplete elementary schooling	72.5	62.8;80.9		24.0	16.8;32.5		45.8	39.2;52.5	
Complete elementary schooling	67.8	57.1;77.2		23.7	15.7;33.4		44.9	37.6;52.3	
Incomplete high school education	54.5	45.6;63.2		25.8	19.0;33.6		39.2	33.5;45.2	
Complete high school education	50.3	45.0;55.6		26.0	21.2;31.3		39.0	35.3;42.8	
Incomplete university education	42.5	36.1;49.1		27.9	21.9;34.6		35.8	31.3;40.4	
Complete university education	33.3	29.0;37.7		18.4	14.3;23.1		27.3	24.2;30.5	
Graduate studies	32.3	27.3;37.5		27.2	20.7;34.6		30.6	26.6;34.8	
SII (%)	40.8 (32.8;48.8)^b^		24.0 (15.5;32.6)^c^	–	–	–	–	–	–
RII	5.5 (3.9;7.9)^b^		2.8 (1.9;4.1)^c^	–	–	–	–	–	–

SII: Slope Index of Inequality; RII: Relative Index of Inequality

^a^ Chi-squared test for linear trend.

^b^ Crude SII and RII.

^c^ Age-adjusted SII and RII.

The education levels were shown to be consistent and inversely associated with abdominal obesity among women, but not among men. Important differences were especially noted among the categories with the same educational attainment. Prevalence of abdominal obesity was higher among women with complete elementary education (67.8%; 95%CI 57.1;77.2) than the one from women with incomplete high school education (54.5%; 95%CI 45.6;63.2). Higher prevalence was found among the women with complete high school education (50.3%; 95%CI 57.1;77.2) than the one from women with incomplete university education (42.5%; 95%CI 45.6;63.2). In the male population, Chi-squared test for linear trend did not rule out the null hypothesis for homogeneity among educational categories (p = 0.436).

SII and RII were only applied to the female population, which is information implying linearity between the polytomous social factor and the health-related outcome.^14^ ^,^ ^16^ ^,^ ^30^ In this sense, the indices of inequality provided a quantitative synthesis of the strictly monotonous trend that is observed between education levels and abdominal obesity (Figure). The numerical score considered the female population as a whole, by making the estimates for inequality regarding abdominal obesity sensitive to population size variations in educational subgroups at different times.

**Figure:**
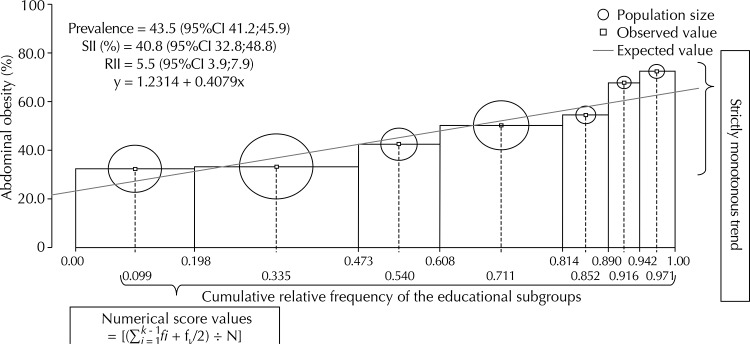
Educational inequality regarding abdominal obesity among multiple educational groups in the female population. *Pró-Saúde* Study, 1999-2001.

The analyses indicated that the consistent and inverse relationship between education levels and abdominal obesity was influenced by the ages; however, it was observed to keep a noticeable pattern (Table 3). The absolute and relative degree of inequality regarding abdominal obesity among multiple educational groups was, respectively, 24.0% (SII; 95%CI 15.5;32.6) and 2.8 (RII; 95%CI 1.9;4.1), after the age adjustment.

The indices of inequality are cross-sectionally correlated in time,^16^ pointing towards the same conclusion about inequality: the probability for being obese was “gradually” higher among the women of lower education levels. However, those indices may describe contradicting results in regards to the degree and direction of educational inequality regarding abdominal obesity throughout time, which comes to highlight the importance of using both measurements.^2^ ^,^ ^14^

## DISCUSSION

The baseline population of EPS was observed to have a high prevalence of abdominal obesity, with an important difference when genders are compared. It was higher among women in all educational strata and age ranges. Gender inequality in the prevalence of abdominal obesity increases with older age and lower education levels, given its steeper direct association with older age in the female population, its inverse one with education levels among women, and not statistically significant association among the men. SII and RII summarized a uniform trend between education levels and abdominal obesity, and they described educational inequality in the occurrence of abdominal obesity among women.

Excess intra-abdominal fat has been particularly important to understand the consequences of obesity. Hypertrophy and hyperplasia of visceral adipocytes increase the risk of hypertriglyceridemia, insulin resistance, and atherosclerosis, regardless of body compositions.^19^ In this sense, abdominal circumference has been observed to have a higher correlation with visceral adipose tissue, as compared to other anthropometric assessments for abdominal adiposity,^4^ ^,^ ^24^ being shown to be more informational when based on an underlying biological argument. Nevertheless, differences regarding measuring techniques and thresholds may influence estimates for prevalence and association with abdominal obesity. A limitation in this study regards to measuring abdominal circumference at the navel level, once the employed thresholds concern measurement at the midpoint between the last rib and the iliac crest.^29^

Socioeconomic position is a complex and multidimensional construct, in which individuals are classified by being compared to other individuals, based on material and non-material attributes.^18^ Frequently used socioeconomic position indicators: education, occupation, and income – each with its advantages and constraints.^11^ ^,^ ^18^ Even though they concern different epidemiological aspects, such indicators have generally pointed towards the same direction in the association with obesity.^1^ ^,^ ^5^ ^,^ ^21^ Notwithstanding, education represents the assets regarding the knowledge of a person,^11^ which assumedly influence ways of living and life styles linked to obesity,^26^ and they also determine other socioeconomic position attributes, such as occupation and income.^11^ ^,^ ^18^

Frequent ways to operationalize education include years of schooling and education attainment. The continuous measurement assumes that each year of schooling similarly contributes to the socioeconomic position, and the categorical measurement assumes that formal education attainment are more relevant for the socioeconomic position than the time one spends with education.^11^

This study considered intermediate levels within a same educational attainment, based on the hypothesis that more time spent with education is relatively important for the association between socioeconomic position and abdominal obesity, which depends on the educational attainment that was reached. That distinction was shown to be important in the distribution of female abdominal obesity in the variation spectrum of education levels.

The indices of inequality provided information that was opportune for longitudinal analyses regarding EPS, as well as for meta-analysis studies and comparisons of the degree of educational inequalities regarding abdominal obesity among populations, geographical areas, and health indicators.^16^ Furthermore, ascribing ordinal values to the categories of polytomous variables may produce quantitatively meaningless dosage-response curves, especially when those categories are internally heterogeneous.^13^

The findings in this research are shown to be in agreement with different cross-sectional and longitudinal studies in distinct populations.^1^ ^,^ ^5^ ^,^ ^21^ ^,^ ^23^ From early socioeconomic development stages, the association between socioeconomic position and obesity becomes predominantly inverse among women, but not among men.^5^ ^,^ ^21^ ^,^ ^23^ In Brazil, time series analyses showed increased prevalence and incidence of obesity, which is associated with lower education only in the female population,^3^ ^,^ ^12^ mainly in urban contexts and the most developed regions in the country.^22^ ^,^ ^a^ A particularly consistent and inverse association between female socioeconomic position and abdominal obesity was found in population-based studies in Rio de Janeiro,^15^ Minas Gerais,^10^ Rio Grande do Sul,^20^ and Maranhao,^27^ especially regarding education, which thus reinforces the proposition of social determination of general and abdominal obesity.

Socioeconomic position influences the individual access to goods and services regarding nutrition, physical activity, and other healthy practices,^26^ as well as environmental conditions which may influence the association between socioeconomic position and abdominal obesity. Groups of higher socioeconomic position tend to eat most nutritious foods, at least partly due to their buying those foods more easily.^6^ They also have higher access to weight-losing methods than groups of lower socioeconomic position.^25^ Besides that, socioeconomic position may cause an impact in attitudes towards one’s own body and in weight-losing practices, especially among women of higher socioeconomic position. They may be more inclined to making efforts towards leaner bodies, whereas obesogenic environments make it harder for women of lower socioeconomic position to do the same.^21^

Among men, the relationship between socioeconomic position and obesity is less clearly observed in medium and high-income contexts. Most studies report not statistically significant associations, the second most frequent result of them being the direct association with income and other indicators of material assets.^5^ ^,^ ^21^ Heavier bodies may be valued differently among genders, and they may represent force and domination for men of higher socioeconomic position.^21^

A rising trend for obesity in association with low socioeconomic position among women in medium and high-income regions will increasingly drive relevant iniquities in different health conditions related to obesity.^5^ ^,^ ^7^ ^,^ ^23^ According to Ezzati et al,^7^ the ‘diseases of affluence’ paradigm must be reconsidered. According to Monteiro et al,^23^ food insecurity and high physical activity patterns became less common after a certain stage of economic development, even for more socially underprivileged segments.

The low percentage of non-respondents (< 5.0%) considerably contributed to the sample representativeness. Regarding the inferential scope, the results in this study do not support generalization to the general population, but they can properly reflect the current patterns in average urban layers of reasonable heterogeneity, as those are an economically active, regularly employed population. No important differences were found when the sociodemographic characteristics of the studied population were compared with the adult population of employed workers in the municipality of Rio de Janeiro.^8^

So far, few studies aimed to examine the relationship between socioeconomic position and abdominal obesity, and not studies were found to describe the degree of educational inequality regarding abdominal obesity by applying indices of inequality in an adult population in Brazil. Potential mediators of that relationship, such as race/ethnicity, area of residence, birth-related cohort, parity, and marital status, need yet to be explored in order to better understand educational inequality regarding abdominal obesity. In conclusion, the indices of inequality provided quantitative estimates that are indispensable for the monitoring of abdominal obesity and for the drafting of public policies.

## Footnotes

a Instituto Brasileiro de Geografia e Estatística. Pesquisa de Orçamentos Familiares 2008-2009: antropometria e estado nutricional de crianças, adolescentes e adultos no Brasil. Rio de Janeiro (RJ); 2010 [cited 2014 Jan 10]. Available from: http://www.ibge.gov.br/home/estatistica/populacao/condicaodevida/pof/2008_2009_encaa/pof_20082009_encaa.pdf
