# Supplementary material for: Educational inequality in the occurrence of abdominal obesity: Pró-Saúde Study
Source: Rev Saude Publica. 2015 Sep 23;49:65. doi: 10.1590/S0034-8910.2015049005786 (PMC4587820; doi:10.1590/S0034-8910.2015049005786)
Supplement: Translation

# Translation: Desigualdade educacional na ocorrência de obesidade abdominal: Estudo Pró-Saúde

**Authors:** Ronaldo Fernandes Santos Alves, Eduardo Faerstein

**Affiliations:** I Programa de Pós-Graduação em Saúde Coletiva. Instituto de Medicina Social. Universidade do Estado do Rio de Janeiro. Rio de Janeiro, RJ, Brasil; II Departamento de Epidemiologia. Instituto de Medicina Social. Universidade do Estado do Rio de Janeiro. Rio de Janeiro, RJ, Brasil

**Keywords:** Obesidade Abdominal, epidemiologia, Fatores Socioeconômicos, Disparidades nos Níveis de Saúde, Gênero e Saúde

## Abstract

**OBJETIVO:**

Estimar o grau de desigualdade educacional na ocorrência de obesidade abdominal em população de funcionários técnico-administrativos de universidade.

**MÉTODOS:**

Neste estudo seccional, utilizamos dados de 3.117 participantes de 24 a 65 anos de idade, de ambos os sexos, referentes à linha de base do Estudo Pró-Saúde, 1999-2001. Obesidade abdominal foi definida segundo pontos de corte para circunferência abdominal de 88 cm para mulheres e 102 cm para homens. Questionário multidimensional autopreenchível foi usado para avaliar escolaridade e variáveis demográficas. Na análise dos dados foram utilizados os índices angular e relativo de desigualdade e o teste de Qui-quadrado para tendência linear. Todas as análises foram estratificadas por gênero e os índices de desigualdade padronizados por idade.

**RESULTADOS:**

Prevalência de obesidade abdominal foi maior entre as mulheres (43,5%; IC95% 41,2;45,9), comparado aos homens (24,3%; IC95% 22,1;26,7), em todos os estratos educacionais e faixas etárias. Associação entre escolaridade e obesidade abdominal foi inversa entre as mulheres (p < 0,001) e estatisticamente não significativa entre os homens (p = 0,436). O grau de desigualdade educacional na obesidade abdominal na população feminina em termos absolutos (Índice Angular de Desigualdade) foi 24,0% (IC95% 15,5;32,6), e em termos relativos (Índice Relativo de Desigualdade) foi 2,8 (IC95% 1,9;4,1), após ajuste por idade.

**CONCLUSÕES:**

A desigualdade de gênero na prevalência de obesidade abdominal aumenta com a idade e menor escolaridade. Os índices angular e relativo de desigualdade resumem a tendência estritamente monotônica entre escolaridade e obesidade abdominal, descrevendo a desigualdade educacional na obesidade abdominal entre as mulheres. Tais índices proporcionam estimativas quantitativas relevantes para o monitoramento da obesidade abdominal e enfrentamento das desigualdades em saúde.

## INTRODUÇÃO

Obesidade é um importante problema de saúde pública global, com tendência crescente em diversos contextos de desenvolvimento.^28^ Pessoas obesas têm maior risco de desfechos adversos em longo prazo, mesmo na ausência de anormalidades metabólicas, quando comparado a indivíduos eutróficos e metabolicamente saudáveis.^17^ No Brasil, aproximadamente 50,0% da população adulta tem excesso de peso e 15,0% tem obesidade.^a^

De acordo com a Organização Mundial da Saúde, obesidade refere-se ao acúmulo anormal ou excessivo de gordura corporal.^28^ Embora o índice de massa corporal tenha sido a medida antropométrica tradicionalmente usada em estudos populacionais, a circunferência abdominal tem apresentado maior capacidade preditiva para diversas morbidades relacionadas à obesidade.^29^ Além disso, por medir a obesidade abdominal, constitui elemento-chave na definição de síndrome metabólica^19^ e na avaliação do risco de doenças cardiovasculares, diabetes, câncer e mortalidade.^4^ ^,^ ^24^

A relação entre posição socioeconômica e obesidade é consistente, mas apresenta variações segundo gênero e nível de desenvolvimento econômico.^5^ ^,^ ^21^ ^,^ ^23^ Em países de renda baixa, verifica-se maior probabilidade de estar obeso entre grupos de alta posição socioeconômica em ambos os sexos. Em países de renda média e alta, a associação entre posição socioeconômica e obesidade mostra-se frequentemente inversa entre as mulheres, principalmente com relação à educação, enquanto assume padrão misto de associação direta e estatisticamente não significativa entre os homens. Essas mudanças no padrão de associação entre posição socioeconômica e obesidade ocorrem em estágios precoces de desenvolvimento econômico, revelando a importância de estudos e intervenções preventivas nesse contexto.^5^ ^,^ ^7^ ^,^ ^21^ ^,^ ^23^

Medidas baseadas em contraste entre dois grupos de comparação são estratégias analíticas comumente utilizadas para reportar magnitude e direção da associação entre posição socioeconômica e obesidade. Contudo, tem como importante desvantagem a interpretação simultânea de diferentes estimativas parciais da desigualdade, uma vez que variáveis politômicas são frequentemente usadas como indicadores de posição socioeconômica.^30^ Métodos alternativos foram propostos para mensuração e monitoramento das desigualdades socioeconômicas em saúde, tendo em vista uma única estimativa quantitativa da desigualdade,^14^ ^,^ ^30^ entretanto estão pouco incorporados à prática da epidemiologia.

Ainda que a relação entre posição socioeconômica e obesidade esteja bem documentada na literatura epidemiológica, sua associação com obesidade abdominal, em particular, ainda é pouco compreendida. Ademais, nenhuma publicação foi encontrada reportando a desigualdade socioeconômica na obesidade abdominal em adultos no Brasil. Explorar a desigualdade relacionada à obesidade abdominal pode ser importante para o aprimoramento de ações de prevenção da obesidade e suas consequências. O objetivo deste estudo foi estimar o grau de desigualdade educacional na ocorrência de obesidade abdominal em população de funcionários técnico-administrativos de universidade.

## MÉTODOS

Neste estudo seccional, utilizamos dados da linha de base do Estudo Pró-Saúde (EPS, 1999-2001). O EPS é um programa de investigação longitudinal de funcionários técnico-administrativos de uma universidade localizada no Rio de Janeiro, com foco principal nos determinantes sociais da saúde.^9^ Até o momento, foram realizadas quatro fases de coleta de dados (1999, 2001, 2006 e 2012) por equipes treinadas, compostas por pesquisadores, supervisores e coordenadores de campo. Participantes elegíveis nas duas primeiras fases, simultaneamente, constituíram a linha de base do EPS.

Incluímos todos os funcionários do quadro efetivo da universidade que aceitaram participar das fases 1 e 2 (linha de base) do EPS. Foram excluídas gestantes e pessoas com mais de 65 anos de idade. Estudo piloto, pré-testes de instrumentos e procedimentos de pesquisa, método de digitação dupla e independente, e monitoramento da coleta e do processamento de dados asseguraram a qualidade das informações analisadas.^8^ ^,^ ^9^ Covariadas foram obtidas do censo de 1999, e as variáveis independente e dependente, em 2001.

A concentração de gordura na região abdominal foi avaliada por meio da circunferência abdominal, aferida em duplicata no nível da cicatriz umbilical, utilizando fita métrica inextensível e inelástica com 180 cm de comprimento. Para aferição, os participantes mantiveram braços cruzados na altura do peito, pés próximos um ao outro, peso distribuído igualmente entre as pernas, abdômen relaxado e respiração normal. Obesidade abdominal foi definida segundo os pontos de corte de 88 cm para mulheres e 102 cm para homens, conforme sugerido pela Organização Mundial da Saúde.^29^

Questionário multidimensional autopreenchível foi utilizado para coleta das informações sobre gênero (masculino; feminino), idade em anos (24 a 34; 35 a 44; 45 a 54; 55 a 65) e escolaridade (fundamental incompleto; fundamental completo; médio incompleto; médio completo; universitário incompleto; universitário completo; pós-graduação).

Análises estatísticas preliminares incluíram: frequências absolutas e relativas, prevalência de obesidade abdominal e os respectivos intervalos de 95% de confiança e o teste de Qui-quadrado para tendência linear entre variáveis ordenadas e desfecho dicotômico. Foram utilizados os índices angular (IAD) e relativo (IRD) de desigualdade para estimar o grau de desigualdade educacional na ocorrência de obesidade abdominal. Esses índices produzem estimativas absolutas e relativas do gradiente socioeconômico na saúde e são baseados em análise de regressão ponderada.^14^ ^,^ ^30^ No cálculo do IAD e do IRD, assim como dos respectivos intervalos de 95% de confiança, usaram-se regressão linear e logística, respectivamente. A variável dependente foi obesidade abdominal (dicotômica). A variável independente foi um escore numérico, definido a partir da mediana do intervalo cumulativo de cada categoria da variável social politômica na escala horizontal da população (Tabela 1). Desse modo, ao invés de atribuir valores ordinais (e.g., 1, 2, 3,..., k, para uma série de k categorias) aos indivíduos das respectivas categorias educacionais, foram atribuídos valores numéricos que consideraram: (a) a informação de todas as categorias ordenadas simultaneamente; (b) o tamanho populacional destas categorias, e (c) a posição relativa das mesmas dentro da escala da população. Por fim, empregaram-se tais valores numéricos, por meio do escore numérico, no modelo de regressão pertinente. O IAD e o IRD foram padronizados por idade.

**Tabela 1:** Medidas de frequência utilizadas na proposição algébrica do escore numérico.

Valores ordinais (variável)	Frequência absoluta	Frequência relativa	Frequência acumulada	Frequência relativa acumulada	Intervalo cumulativo da população	Mediana do intervalo (escore)
1	f_1_	fr_1_	F_1_	Fr_1_	0,0 - Fr_1_	Escore 1
2	f_2_	fr_2_	F_2_	Fr_2_	Fr_1_ |-Fr_2_	Escore 2
3	f_3_	fr_3_	F_3_	Fr_3_	Fr_2_ |-Fr_3_	Escore 3
...	...	...	...	...	...	...
k	f_k_	fr_k_	F_k_ = N	Fr_k_ = 1,0	Fr_k-1_ |-1,0	Escore k

k: índice (ordinal) da categoria da variável social politômica; f_k_: frequência absoluta; fr_k_: frequência relativa (expressa comumente em %); F_k_: frequência acumulada; Fr_k_: frequência relativa acumulada; N: total de indivíduos da população

Nota: [fr_k_ = f_k_ ÷ N] e [Fr_k_ = F_k_ ÷ N]; [f_1_ = F_1_] e [fr_1_ = Fr_1_].

Abaixo está uma proposição algébrica para elucidar o escore numérico. Na Tabela 1, tem-se uma disposição lógica em formato matricial das medidas de frequência utilizadas, a fim de apoiar a leitura da formulação do escore numérico.





Todas as análises foram estratificadas por gênero e transcorreram no *software* R 3.1.0.

Os protocolos do EPS 1999 e 2001 foram aprovados pelo Comitê de Ética em Pesquisa do Hospital Universitário Pedro Ernesto da Universidade do Estado do Rio de Janeiro (Registro 224/1999; Registro 461/2001). Todos os participantes assinaram termo de consentimento livre e esclarecido. Os dados foram analisados de maneira a garantir o sigilo dos participantes.

## RESULTADOS

A população de estudo foi composta por participantes elegíveis nas duas primeiras fases do EPS, 1999 e 2001. Durante o trabalho de campo, houve 9,6% de não participação na fase 1, bem como 16,5% na fase 2, totalizando 3.253 participantes em ambas as fases (77,9% dos elegíveis), os quais referem-se à linha de base do programa de investigação. Não foi possível aferir o perímetro abdominal em 52 pessoas (1,6%); 21 participantes (0,7%) foram excluídos da análise de dados por terem mais de 65 anos de idade; 63 participantes (1,9%) não responderam à pergunta relativa à variável escolaridade, obtendo-se por fim uma amostra de 3.117 adultos (95,8% dos participantes).

A amostra foi constituída por uma proporção ligeiramente maior de mulheres (Tabela 2). A média de idade foi 42,7 (IC95% 42,3;43,1) anos para as mulheres e 41,2 (IC95% 40,8;41,6) para os homens. As mulheres apresentaram maior nível educacional, sendo que 47,0% possuíam pelo menos o ensino superior completo, enquanto para os homens a proporção foi de 36,0%. A média de idade foi maior nas categorias de menor escolaridade, variando de 53 a 40 anos entre as mulheres e de 48 a 40 anos entre os homens.

**Tabela 2:** Características sociodemográficas da linha de base do Estudo Pró-Saúde, 1999-2001.

Variável	Mulheres		Homens		Total	
	n	%	n	%	N	%
Sexo	1.739	55,8	1.378	44,2	3.117	100
Idade (anos)						
24 a 34	281	16,2	307	22,3	588	18,9
35 a 44	765	44,0	623	45,2	1.388	44,5
45 a 54	541	31,1	367	26,6	908	29,1
55 a 65	152	8,7	81	5,9	233	7,5
Escolaridade						
Fundamental incompleto	102	5,9	125	9,1	227	7,3
Fundamental completo	90	5,2	97	7,0	187	6,0
Médio incompleto	132	7,6	151	11,0	283	9,1
Médio completo	358	20,6	311	22,6	669	21,5
Superior incompleto	235	13,5	204	14,8	439	14,1
Superior completo	478	27,5	321	23,3	799	25,6
Pós-graduação	344	19,8	169	12,3	513	16,5

A prevalência de obesidade abdominal na população de linha de base do EPS foi 35,0% (IC95% 33,3;36,7), sendo significativamente maior entre as mulheres (43,5%; IC95% 41,2;45,9), em comparação aos homens (24,3%; IC95% 22,1;26,7), em todos os estratos educacionais e faixas etárias (Tabela 3). Entre as mulheres, a probabilidade de estar obeso aumentou conforme a idade e foi marcadamente maior no grupo de 55 a 65 anos (73,0%; IC95% 65,2;79,9). Observou-se declínio da prevalência de obesidade abdominal entre os homens de 55 a 65 anos, embora seja possível que o tamanho populacional reduzido deste subgrupo etário tenha influenciado a estimativa pontual.

**Tabela 3:** Prevalência (%) de obesidade abdominal, índice angular de desigualdade e índice relativo de desigualdade da população feminina. Estudo Pró-Saúde, 1999-2001.

Variável	Mulheres			Homens			Total		
	Obesidade abdominal (%)	IC95%	p^a^	Obesidade abdominal (%)	IC95%	p^a^	Obesidade abdominal (%)	IC95%	p^a^
Sexo	43,5	41,2;45,9		24,3	22,1;26,7		35,0	33,3;36,7	
Idade (anos)			< 0,001			< 0,001			< 0,001
24 a 34	26,0	20,9;31,5		16,9	12,9;21,6		21,3	18,0;24,8	
35 a 44	36,3	32,9;39,9		24,1	20,8;27,6		30,8	28,4;33,3	
45 a 54	54,5	50,2;58,8		30,2	25,6;35,2		44,7	41,4;48,0	
55 a 65	73,0	65,2;79,9		27,2	17,9;38,2		57,1	50,4;63,5	
Escolaridade			< 0,001			0,436			< 0,001
Fundamental incompleto	72,5	62,8;80,9		24,0	16,8;32,5		45,8	39,2;52,5	
Fundamental completo	67,8	57,1;77,2		23,7	15,7;33,4		44,9	37,6;52,3	
Médio incompleto	54,5	45,6;63,2		25,8	19,0;33,6		39,2	33,5;45,2	
Médio completo	50,3	45,0;55,6		26,0	21,2;31,3		39,0	35,3;42,8	
Superior incompleto	42,5	36,1;49,1		27,9	21,9;34,6		35,8	31,3;40,4	
Superior completo	33,3	29,0;37,7		18,4	14,3;23,1		27,3	24,2;30,5	
Pós-graduação	32,3	27,3;37,5		27,2	20,7;34,6		30,6	26,6;34,8	
IAD (%)	40,8 (32,8;48,8)^b^		24,0 (15,5;32,6)^c^	–	–	–	–	–	–
IRD	5,5 (3,9;7,9)^b^		2,8 (1,9;4,1)^c^	–	–	–	–	–	–

IAD: Índice Angular de Desigualdade; IRD: Índice Relativo de Desigualdade

^a^ teste Qui-quadrado para tendência linear.

^b ^IAD e IRD brutos.

^c^ IAD e IRD ajustados por idade.

Escolaridade mostrou-se consistente e inversamente associada à obesidade abdominal entre as mulheres, mas não entre os homens. Em particular, notaram-se diferenças importantes entre as categorias de mesmo patamar educacional. A prevalência de obesidade abdominal foi maior entre mulheres com ensino fundamental completo (67,8%; IC95% 57,1;77,2) do que entre mulheres com ensino médio incompleto (54,5%; IC95% 45,6;63,2). Encontrou-se prevalência maior entre mulheres com ensino médio completo (50,3; IC95% 45,0;55,6) em comparação àquelas com ensino superior incompleto (42,5%; IC95% 36,1;49,1). Na população masculina, o teste de Qui-quadrado para tendência linear não rejeitou a hipótese nula de homogeneidade entre as categorias educacionais (p = 0,436).

IAD e IRD foram aplicados somente à população feminina, dado que pressupõem linearidade entre o fator social politômico e o desfecho de saúde.^14^ ^,^ ^16^ ^,^ ^30^ Nesse sentido, os índices de desigualdade proporcionaram uma síntese quantitativa da tendência estritamente monotônica observada entre escolaridade e obesidade abdominal (Figura). O escore numérico considerou a experiência da totalidade da população feminina, tornando as estimativas de desigualdade na obesidade abdominal sensíveis a variações temporais no tamanho populacional dos subgrupos educacionais.

**Figura:** Desigualdade na ocorrência de obesidade abdominal entre os múltiplos grupos educacionais na população feminina. EPS, 1999-2001.

As análises indicaram que a relação consistente e inversa entre escolaridade e obesidade abdominal sofreu influência da idade, porém manteve padrão notável (Tabela 3). A magnitude absoluta e relativa da desigualdade na obesidade abdominal entre os múltiplos grupos educacionais foi, respectivamente, 24,0% (IAD; IC95% 15,5;32,6) e 2,8% (IRD; IC95% 1,9;4,1), após ajuste por idade.

Os índices de desigualdade estão correlacionados transversalmente no tempo,^16^ apontando para a mesma conclusão acerca da desigualdade: a probabilidade de estar obeso foi ‘gradativamente’ maior entre as mulheres de menor escolaridade. Contudo, estes índices podem descrever resultados discordantes quanto à magnitude e direção da desigualdade educacional na obesidade abdominal ao longo do tempo, salientando a importância da utilização de ambas as medidas.^2^ ^,^ ^14^

## DISCUSSÃO

A população de linha de base do EPS apresentou elevada prevalência de obesidade abdominal, com importante diferença entre os gêneros, sendo maior entre as mulheres em todos os estratos educacionais e etários. A desigualdade de gênero na prevalência de obesidade abdominal aumentou conforme a idade e menor educação, dada associação direta com idade mais acentuada na população feminina e inversa com escolaridade entre as mulheres e estatisticamente não significativa entre os homens. IAD e IRD resumiram uma associação uniforme entre escolaridade e obesidade abdominal, descrevendo a desigualdade educacional na ocorrência de obesidade abdominal entre as mulheres.

O excesso de gordura intra-abdominal tem sido particularmente importante na compreensão das consequências da obesidade. A hipertrofia e hiperplasia de adipócitos viscerais aumentam o risco de hipertrigliceridemia, resistência à insulina e aterosclerose, independentemente da composição corporal.^19^ Neste sentido, a circunferência abdominal tem apresentado maior correlação com tecido adiposo visceral, quando comparada a outras medidas antropométricas de adiposidade abdominal,^4^ ^,^ ^24^ mostrando-se mais informativa com base em argumento biológico subjacente. Entretanto, diferenças quanto à técnica de aferição e ponto de corte podem influenciar estimativas de prevalência e associação para com obesidade abdominal. Uma limitação deste estudo concerne à aferição da circunferência abdominal no nível da cicatriz umbilical, uma vez que os pontos de corte empregados referem-se à mensuração no ponto médio entre a última costela e a crista ilíaca.^29^

Posição socioeconômica é um construto complexo e multidimensional em que indivíduos são classificados em relação a outros indivíduos, baseado em atributos materiais e não materiais.^18^ Indicadores de posição socioeconômica, frequentemente utilizados, incluem: escolaridade, ocupação e renda, cada qual com suas vantagens e limitações.^11^ ^,^ ^1^ ^8^ Ainda que confiram diferentes aspectos de interesse epidemiológico, tais indicadores têm apontado geralmente para a mesma direção na associação com obesidade.^1^ ^,^ ^5^ ^,^ ^21^ Não obstante, escolaridade representa os ativos relacionados ao conhecimento de uma pessoa^11^ que presumidamente influenciam modos e estilos de vida relacionados à obesidade,^26^ assim como determinam outros atributos da posição socioeconômica, como ocupação e renda.^11^ ^,^ ^18^

Maneiras frequentes de operacionalizar escolaridade incluem anos de estudos e patamares educacionais. A medida contínua pressupõe que cada ano de estudo contribui de maneira semelhante para a posição socioeconômica e a medida categórica assume que patamares de educação formal são mais relevantes para posição socioeconômica que o tempo gasto com educação.^11^

O presente estudo considerou níveis intermediários dentro de um mesmo patamar educacional, baseado na hipótese que maior tempo desprendido com educação é relativamente importante para associação entre posição socioeconômica e obesidade abdominal, condicional ao patamar de educação alcançado. Esta discriminação mostrou-se importante na distribuição da obesidade abdominal feminina no espectro de variação da escolaridade.

Os índices de desigualdade forneceram informação oportuna para análises longitudinais no âmbito do EPS, assim como para estudos de meta-análise e comparações da magnitude da desigualdade educacional na obesidade abdominal entre populações, áreas geográficas e indicadores de saúde.^16^ Adicionalmente, atribuir valores ordinais para as categorias de variáveis politômicas pode produzir curvas de dose-resposta quantitativamente sem sentido, em especial quando estas categorias forem heterogêneas internamente.^13^

Os achados desta pesquisa mostram concordância com diferentes estudos seccionais e longitudinais, em populações distintas.^1^ ^,^ ^5^ ^,^ ^21^ ^,^ ^23^ A partir de estágios iniciais de desenvolvimento socioeconômico, a associação entre posição socioeconômica e obesidade torna-se predominantemente inversa entre as mulheres, mas não entre os homens.^5^ ^,^ ^21^ ^,^ ^23^ No Brasil, análises temporais evidenciaram aumento da prevalência e incidência de obesidade, associado à menor escolaridade apenas na população feminina,^3^ ^,^ ^12^ principalmente em contextos urbanos e regiões mais desenvolvidas do País.^22^ ^,^ ^a^ Associação consistente e inversa entre posição socioeconômica e obesidade abdominal feminina, em particular, foi encontrada em estudos de base populacional no Rio de Janeiro,^15^ Minas Gerais,^10^ Rio Grande do Sul^20^ e Maranhão,^27^ principalmente com relação à escolaridade, reforçando a proposição de determinação social da obesidade geral e abdominal.

A posição socioeconômica influencia o acesso individual a bens e serviços relativos à alimentação, atividade física e outras práticas saudáveis,^26^ assim como condições ambientais que podem influenciar a associação entre posição socioeconômica e obesidade abdominal. Grupos de maior posição socioeconômica tendem a consumir alimentos de melhor qualidade nutricional, devido, ao menos em parte, ao maior poder de adquirir esses alimentos,^6^ bem como tem maior acesso aos meios de redução voluntária do peso corporal que grupos de menor posição socioeconômica.^25^ Além disso, a posição socioeconômica pode exercer impacto em atitudes em torno do próprio corpo e em práticas de controle do peso corporal, especialmente entre as mulheres de maior posição socioeconômica. Estas podem estar mais expostas a valorizar e buscar uma estética de corpo esbelto, ao passo que o ambiente obesogênico torna mais difícil fazê-lo para as mulheres de mais baixa posição socioeconômica.^21^

Entre os homens, a relação entre posição socioeconômica e obesidade manifesta-se de forma menos clara em contextos de renda média e alta. A maioria dos estudos reporta associação estatisticamente não significativa, sendo o segundo resultado mais frequente a associação direta com renda e outros indicadores de recursos materiais.^5^ ^,^ ^21^ É possível que a corpulência tenha valor simbólico diferenciado entre os sexos, podendo representar força e dominação para os homens de maior posição socioeconômica.^21^

Tendência crescente de obesidade associada à baixa posição socioeconômica entre as mulheres de regiões de renda média e alta, cada vez mais impulsionará relevantes iniquidades em diferentes condições de saúde relacionadas à obesidade;^5^ ^,^ ^7^ ^,^ ^23^ o que, segundo Ezzati et al,^7^ sugere repensar o paradigma de doença da afluência. De acordo com Monteiro et al,^23^ padrões de insegurança alimentar e de elevado gasto energético tornaram-se menos comuns depois de certo estágio de desenvolvimento econômico, mesmo para os segmentos em maior desvantagem social.

O baixo percentual de não respondentes (< 5,0%) contribuiu consideravelmente para representatividade da amostra. A respeito do escopo inferencial, os resultados deste estudo não apoiam generalização para população geral, mas podem refletir adequadamente os padrões vigentes em camadas médias urbanas com razoável heterogeneidade, por tratar-se de uma população economicamente ativa e com vínculo empregatício estável. Comparando as características sociodemográficas da população de estudo com a população adulta de trabalhadores ocupados no município do Rio de Janeiro, não foram verificadas diferenças importantes.^8^

Até o momento, poucos estudos buscaram examinar a relação entre posição socioeconômica e obesidade abdominal, e nenhum estudo foi encontrado descrevendo o grau de desigualdade educacional na ocorrência de obesidade abdominal, aplicando os índices de desigualdade, em população adulta no Brasil. Potenciais mediadores desta relação, tais como raça/etnia, área de residência, coorte de nascimento, paridade e situação conjugal, ainda precisam ser explorados para melhor compreender a desigualdade educacional na obesidade abdominal. Em conclusão, os índices de desigualdade forneceram estimativas quantitativas indispensáveis ao monitoramento da obesidade abdominal e à elaboração de políticas públicas voltadas para redução das desigualdades em saúde.

## Footnotes

a Instituto Brasileiro de Geografia e Estatística. Pesquisa de Orçamentos Familiares 2008-2009: antropometria e estado nutricional de crianças, adolescentes e adultos no Brasil. Rio de Janeiro (RJ); 2010 [citado 2014 jan 10]. Disponível em: http://www.ibge.gov.br/home/estatistica/populacao/condicaodevida/pof/2008_2009_encaa/pof_20082009_encaa.pdf
